# Potential prognostic value of a eight ferroptosis-related lncRNAs model and the correlative immune activity in oral squamous cell carcinoma

**DOI:** 10.1186/s12863-022-01097-z

**Published:** 2022-11-16

**Authors:** Lin Qiu, Anqi Tao, Fei Liu, Xianpeng Ge, Cuiying Li

**Affiliations:** 1 Central Laboratory, Peking University School and Hospital of Stomatology& National Center of Stomatology & National Clinical Research Center for Oral Diseases & National Engineering Research Center of Oral Biomaterials and Digital Medical Devices, Beijing, China; 2 Department of Dentistry, Xuanwu Hospital Capital Medical University, Beijing, China; 3 National Clinical Research Center for Geriatric Disorders, Beijing, China

**Keywords:** Oral squamous cell carcinoma, Ferroptosis, Long non-coding RNAs, Immune activity

## Abstract

**Background:**

To investigate the prognostic value of ferroptosis-related long noncoding RNAs (lncRNAs) in oral squamous cell carcinoma (OSCC) and to construct a prognostic risk and immune activity model.

**Methods:**

We obtained clinical and RNA-seq information on OSCC patient data in The Cancer Genome Atlas (TCGA) Genome Data Sharing (GDC) portal. Through a combination of a differential analysis, Pearson correlation analysis and Cox regression analysis, ferroptosis-related lncRNAs were identified, and a prognostic model was established based on these ferroptosis-related lncRNAs. The accuracy of the model was evaluated via analyses based on survival curves, receiver operating characteristic (ROC) curves, and clinical decision curve analysis (DCA). Univariate Cox and multivariate Cox regression analyses were performed to evaluate independent prognostic factors. Then, the infiltration and functional enrichment of immune cells in high- and low-risk groups were compared. Finally, certain small-molecule drugs that potentially target OSCC were predicted via use of the L1000FWD database.

**Results:**

The prognostic model included 8 ferroptosis-related lncRNAs (FIRRE, LINC01305, AC099850.3, AL512274.1, AC090246.1, MIAT, AC079921.2 and LINC00524). The area under the ROC curve (AUC) was 0.726. The DCA revealed that the risk score based on the prognostic model was a better prognostic indicator than other clinical indicators. The multivariate Cox regression analysis showed that the risk score was an independent prognostic factor for OSCC. There were differences in immune cell infiltration, immune functions, m6A-related gene expression levels, and signal pathway enrichment between the high- and low-risk groups. Subsequently, several small-molecule drugs were predicted for use against differentially expressed ferroptosis-related genes in OSCC.

**Conclusions:**

We constructed a new prognostic model of OSCC based on ferroptosis-related lncRNAs. The model is valuable for prognostic prediction and immune evaluation, laying a foundation for the study of ferroptosis-related lncRNAs in OSCC.

**Supplementary Information:**

The online version contains supplementary material available at 10.1186/s12863-022-01097-z.

## Introduction

Oral cancer ranks among the most prevalent malignant tumours in the head and neck. In 2020, more than 350,000 newly confirmed cases and 175,000 deaths from oral cancers were reported worldwide [1]. Oral squamous cell carcinoma (OSCC) accounts for 90% of oral cancers [2]. At present, many clinical guidelines clearly indicate that the diagnosis and treatment of OSCC cannot be generalized, and the use of comprehensive sequence therapy should be accompanied by individualized treatment [3]. However, despite this guidance, a OSCC diagnosis is a poor prognosis, with a 5-year survival rate of approximately 60% [4]. OSCC is also associated with a high cervical lymph node metastasis rate, leading to a worsened prognosis [5]. Therefore, finding new predictors of survival and developing new detection methods for better clinical decision-making are essential.

Ferroptosis refers to an iron-dependent cell death process, and the morphological characteristics and biochemical markers of ferroptosis are significantly different from those of apoptosis, necrosis, and autophagy [6,7]. Although ferroptosis was first described in 2012 [8], a clearer understanding of ferroptosis-related mechanisms and functions have since led researchers to show that ferroptosis is inseparable from tumours. Recent research has revealed the association of ferroptosis with tumorigenesis and progression in, for example, bladder cancer [9], ovarian cancer [10] and breast cancer [11]. In addition, ferroptosis plays a role in tumours by interacting with different components in the tumour microenvironment (TME). Tumour cells with reduced E-cadherin levels and loss of intercellular adhesion have been reported to be highly sensitive to ferroptosis [12,13], and cell density is an important factor in determining the susceptibility to ferroptosis regardless of the cell-specific phenotype [14]. Most solid tumours are hypoxic, and hypoxia increases the level of carbonic anhydrase 9 (CA9). Studies have shown that elevated CA9 can reduce ferroptosis by controlling intracellular iron metabolism [15]. Ferroptosis also affects tumour cell sensitivity to radiotherapy and can be used to overcome chemotherapy resistance [16,17]. In OSCC, certain ferroptosis-related genes, such as SLC7A11 [18] and GPX4 [19], can impact the prognosis of patients by regulating ferroptosis in cancer cells. These findings suggest that developing ferroptosis-related treatment strategies is an emerging direction for OSCC treatment.

Long noncoding RNAs (lncRNAs) are RNAs with a transcript length between 200 and 100,000 nt and that do not encode proteins but participates in many physiological processes [20]. To date, more than 1 × 10^6^ lncRNAs have been reported in the human genome, and it has been indicated that disordered lncRNAs are closely connected to the occurrence and development of human cancers [21]. LncRNAs can regulate biological behaviours such as tumour cell proliferation, apoptosis, invasion, and metastasis. Recently, the effects of lncRNAs on ferroptosis regulation have been studied by researchers. Studies have shown that lncRNAs, as dual regulators of ferroptosis, either participate in ferroptosis by inactivating certain miRNAs, as endogenous competing RNAs, or binding to certain enzymes to regulate ferroptosis and influence the biological activity of cancer cells [22]. The most recent reports revealed an association of ferroptosis-related lncRNAs with the prognosis of various cancers, such as colon adenocarcinoma [23] and breast cancer [24]. However, the role played by ferroptosis as well as its associated lncRNAs in OSCC remains unclear. Therefore, studying lncRNAs associated with OSCC and ferroptosis is crucial for understanding the mechanisms underlying OSCC.

Bioinformatics techniques constitute a new technological approach by effectively combining bioinformatics with medicine. Functional genomics based on bioinformatics is a rapidly developing field [25]. The TCGA database includes complete genome-sequencing studies of a variety of tumours, providing great help for scientific research and discovery of new molecular targets in tumours. Many tumour biomarkers have been discovered and applied clinically, significantly leading to early diagnosis of tumours and increasing the overall survival rate [26,27]. Recently, a model containing 8 ferroptosis-related lncRNAs has been reported; however, the model exhibited low predictive power for OSCC, with an area under the curve (AUC) = 0.690 [28]. Other scholars constructed a prognostic model containing 9 ferroptosis-related lncRNAs [29], which only used bioinformatics to explore the relationship between ferroptosis-related lncRNAs and the prognosis of head and neck squamous cell carcinoma patients. In addition, this model was not specific for OSCC and lacks relevant in vitro experimental validation. A new prognostic model of OSCC incorporating ferroptosis-related lncRNAs was developed using bioinformatics methods. The prognostic ability of this model was confirmed, and immune function was analysed via different methods. In addition, we investigated differentially expressed ferroptosis genes in the L1000FWD database, identifying small-molecule drugs that potentially target ferroptosis genes in OSCC.

## Materials and methods

### Data collection

**Fig. 1:**
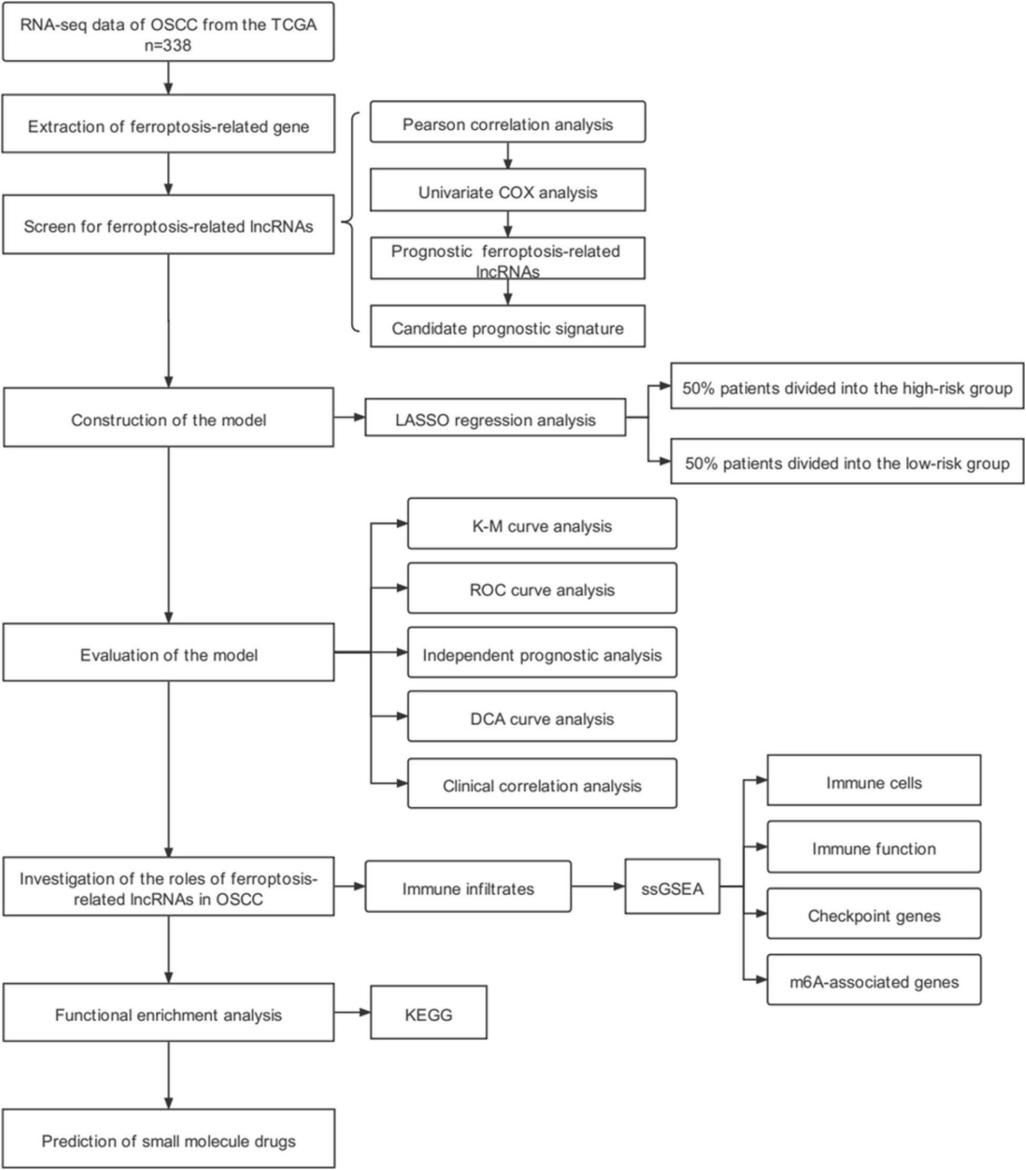
Study design flowchart

We obtained RNA sequencing (FPKM) and clinical information on OSCC from the TCGA (https://portal.gdc.cancer.gov/). Table 1 presents the clinical data for 338 samples. According to the FerrDb website (http://www.zhounan.org/ferrdb/) and previous research, 382 ferroptosis-related genes were identified, including ferroptosis-inducing genes, ferroptosis-suppressing genes and ferroptosis markers. The codes used in this study can be found on Github (https://github.com/qiulin961028/ferroptosis-related-lncRNAs), and Fig. 1 shows the flow chart.

**Table 1:** Clinical features of TCGA-OSCC patients

Characteristic	*N* = 338	
Age	Median	61
	Range	19–88
Sex	Male	232
	Female	106
Grade	G1	52
	G2	207
	G3	65
	G4	4
	NA	10
Clinical stage	Stage I	22
	Stage II	57
	Stage III	62
	Stage IV	161
	NA	36
T stage	T1	36
	T2	105
	T3	69
	T4	100
	NA	28
M stage	M0	121
	M1	0
	NA	217
N stage	N0	121
	N1	51
	N2	107
	N3	4
	NA	55
Vital status	Alive	187
	Dead	151

### Construction and validation of the prognostic model

Ferroptosis-related gene expression was determined for the samples, and Pearson correlation analysis was performed to identify ferroptosis-related lncRNAs (|correlation coefficient|> 0.4, *p* < 0.001). Then, we acquired lncRNAs that show prognostic promise in ferroptosis as determined through univariate Cox regression (*p* < 0.05). Before establishing the model, we constructed a network with ferroptosis-related mRNAs and lncRNAs, followed by visualization using Cytoscape. The prognostic risk model was further refined by multivariate Cox regression analysis, and the risk score for patients was calculated using Eq. (1):

1 $${\varvec{R}}{\varvec{i}}{\varvec{s}}{\varvec{k}}{\varvec{s}}{\varvec{c}}{\varvec{o}}{\varvec{r}}{\varvec{e}}={\sum }_{{\varvec{i}}=1}^{{\varvec{n}}}{\varvec{C}}{\varvec{o}}{\varvec{e}}{\varvec{f}}{\varvec{i}}\times {\varvec{X}}{\varvec{i}}$$

*Coefi* is the risk regression coefficient for every ferroptosis-related lncRNA, and *X* represents the lncRNA expression level. Based on this model, patients’ risk scores were measured, and the patients were assigned to a low- or high-risk group in with the median risk score serving as the cut-off value.

Immediately afterward this analysis, the overall survival (OS) for patients with OSCC was compared between the two risk groups via a survival analysis. The accuracy of the prognostic model was evaluated on the basis of ROC curves. We thus identified factors that independently predicted prognosis via univariate and multifactorial Cox regression. Prognostic correlation line graphs including age, risk score, sex, tumour grade, and TN stage were plotted with the "RMS" package in R language software, and internal calibration curves were plotted for the line graphs. LNCipedia (https://lncipedia.org/) was used to retrieve ferroptosis-related lncRNA sequences, and the lncLocator database (http://www.csbio.sjtu.edu.cn/bioinf/lncLocator/) was used to identify lncRNA cellular compartment localization based on its sequence.

### Immune cell infiltration prediction

To evaluate the degree of immune cell infiltration, we performed a ssGSEA to quantify subgroups of infiltrating immune cells in conjunction with the immune function of both groups. The underlying immune checkpoint and m6A genes were identified based on previous research, and gene expression differences between the two groups were examined.

### Pathway enrichment analysis

Further, Kyoto Encyclopedia of Genes and Genomes (KEGG) analyses were performed with both groups. Using GSEA (4.1.1) software, the data were analysed, and enrichment maps were created.

### Potential small molecule drug prediction

Differentially expressed ferroptosis-related genes were classified into up- or downregulated groups and imported into the L1000FWD website (https://maayanlab.cloud/L1000FWD/) to obtain permuted outcomes. Drug structures are shown on PubChem.ncbi.nlm.nih.gov.

### Cell culture

Human OSCC cell lines WSU-HN6 and CAL-27 were used in this study. WSU-HN6 was obtained from Ninth People's Hospital, Shanghai Jiao Tong University School of Medicine (Shanghai, China), and CAL-27 cell line was purchased from American Type Culture Collection (ATCC, Manassas, USA). All cells were passaged and preserved in the Central Laboratory of Peking University Hospital of Stomatology and regularly tested to ensure mycoplasma negative. All cells were cultured in high glucose DMEM medium (Gibco, CA, USA) containing 10% fetal bovine serum (Gibco, CA, USA) and 1% penicillin/streptomycin solution at 37 °C and 5% CO_2_.

### Real-time PCR

Total RNA was extracted from cells and tissues using Trizol. Cytoplasmic and nuclear RNA were isolated and purified using the Nuc-Cyto-Mem Preparation Kit (APPLYGEN) and Trizol according to the manufacturers' instructions. Then totol RNA reverse transcribed into cDNA using a Prime Script™ RT Kit. The cDNA template was subsequently amplified by real-time PCR (RT‒PCR) using SYBR Green qPCR Master Mix (ABclonal, Beijing, China). GAPDH and U6 wer used as the internal reference and mRNA relative expression was measured by the 2^−ΔΔCT^ method. The primer sequences were shown in supplementary Table 1.

### Statistical analysis

For gene expression levels, the Wilcoxon test and unpaired Student's t test were performed with data showing with a normal and a nonnormal distribution, respectively. We assessed OSCC patient survival by Kaplan‒Meier curves, and ROC analysis and DCA were performed with the "timeROC" and "ggDCA" software packages, respectively. Data analysis was performed using R software (4.1.1), with *P* < 0. 05 indicating a significant difference.

## Results

### Data processing and discovery of ferroptosis-associated lncRNAs with prognostic significance

**Fig. 2:**
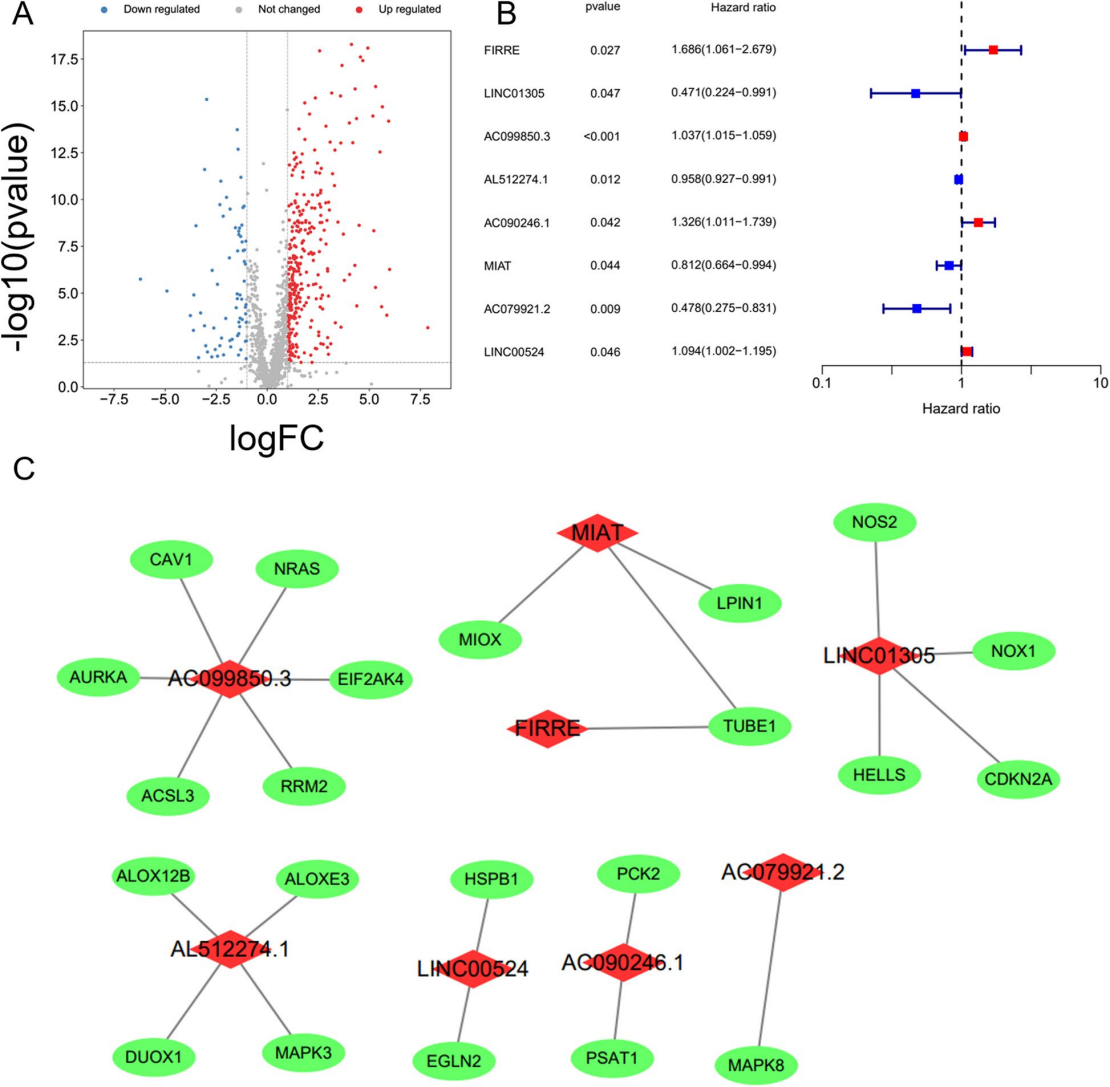
Data collection and analysis. A Volcano plot showing differentially expressed lncRNAs; blue points indicate a logFC < -1, red points indicate a logFC > 1, *p* < 0. 05. B Forest plot of prognosis-related differentially expressed lncRNAs. C Visualization of the network that contained ferroptosis-associated mRNAs and lncRNAs by Cytoscape. Green and red nodes represent ferroptosis-associated mRNAs and lncRNAs, respectively

A total of 386 differentially expressed lncRNAs in OSCC were obtained by rank sum test (Fig. 2A). We obtained differentially expressed ferroptosis-related lncRNAs via correlation analysis, and eight ferroptosis- and prognosis-related lncRNAs were recognized via univariate Cox survival analysis: FIRRE, LINC01305, AC099850.3, AL512274.1, AC090246.1, MIAT, AC079921.2 and LINC00524 (Fig. 2B). A correlation network between ferroptosis genes and these prognosis-related lncRNAs was constructed and visualized by Cytoscape (Fig. 2C). Among these lncRNAs, AC099850.3, LINC01305, and AL512274.1 were coexpressed with a relatively higher number of ferroptosis genes.

### Prognostic model establishment and verification

A prognostic risk model was established on the basis of Cox regression analysis; then, we determined risk scores for all cases for the expression levels of risk regression coefficients and ferroptosis-related lncRNAs. Risk score = [FIRRE expression × (0.66714)] + [LINC01305 expression × (0.78751)] + [AC099850.3 expression × (0.029993)] + [AL512274.1 expression × (0.05794)] + [AC090246.1 expression × (0.541331)] + [MIAT expression × (0.24386)] + [AC079921.2 expression × (0.75098)] + LINC00524 expression × (0.105386)].

**Fig. 3:**
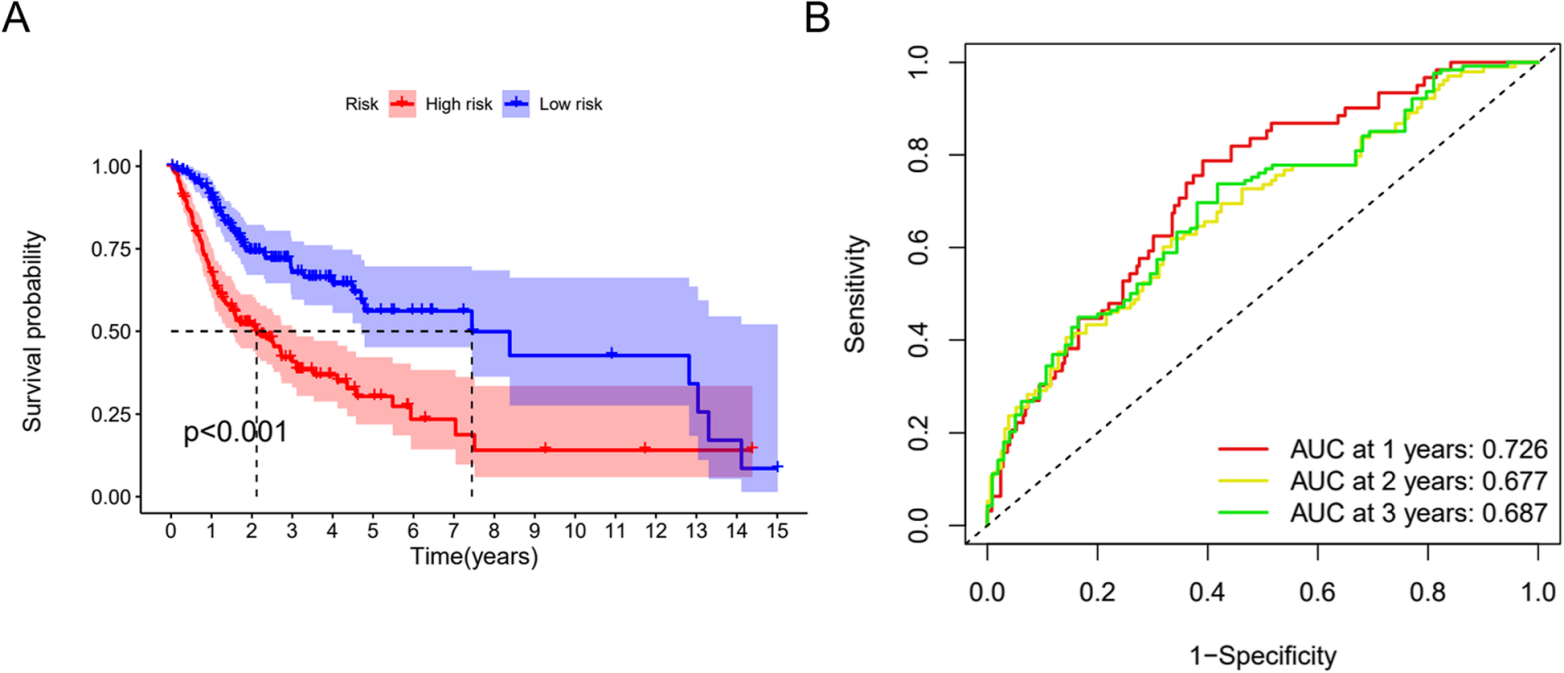
Construction and validation of the risk model. A Kaplan‒Meier analysis of the risk model for both groups. B ROC curves and AUC values at 1, 2 and 3 years

The survival analysis results revealed an the obviously lower OS rate in the high-risk group compared with that in the low-risk group (*p* < 0.001) (Fig. 3A). The ROC curve showed 1-, 2- and 3-year area under the curve (AUC) values of 0.726, 0.677, and 0.687, respectively (Fig. 3B), suggesting that the risk model showed good performance for predicting patient prognosis.

### The risk score independently predicts OSCC prognosis

**Fig. 4:**
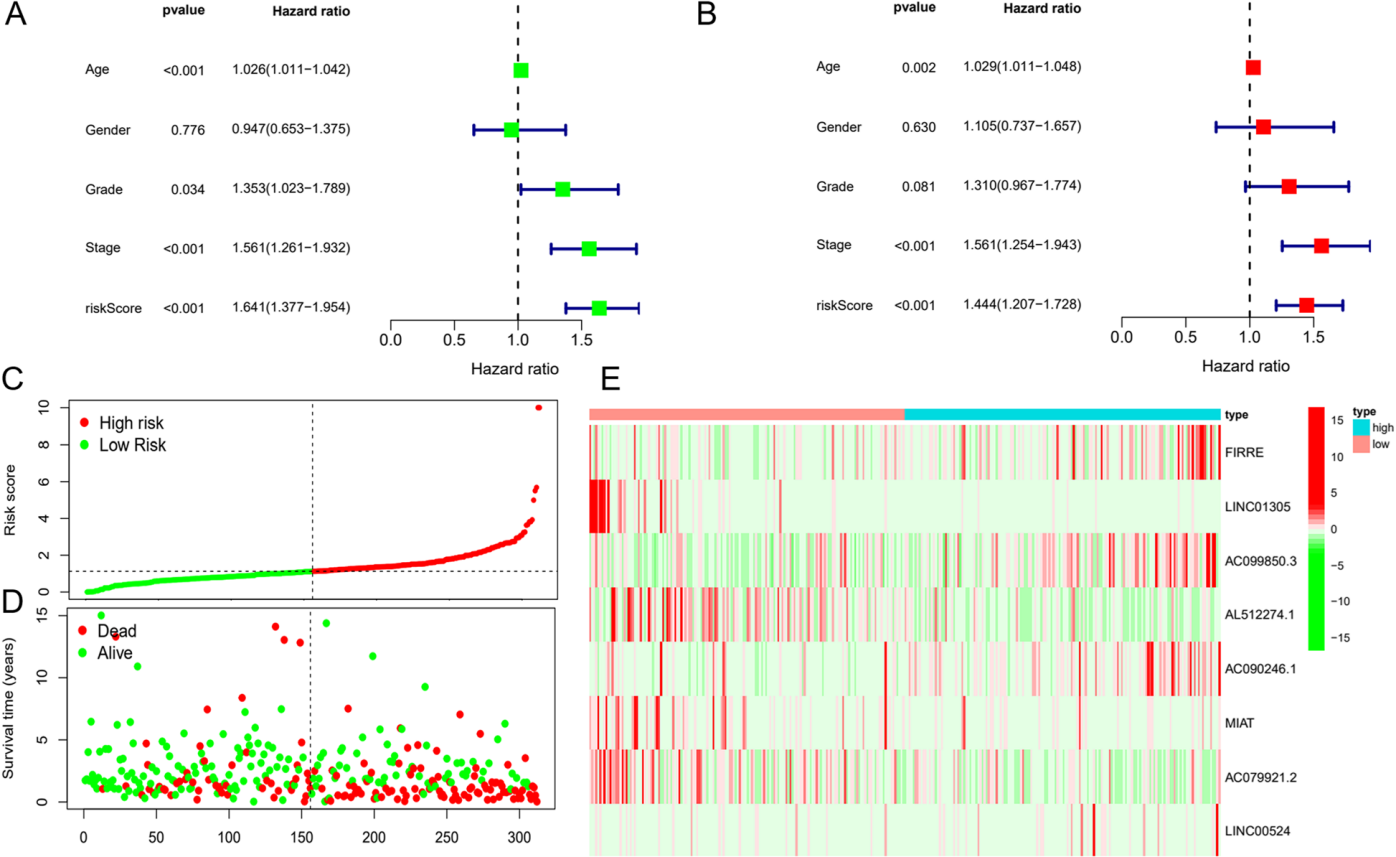
Evaluation of the feasibility of the risk score to independently predict OSCC prognosis. A Univariate Cox regression; *p* < 0. 05 indicates statistical significance. B Multivariate Cox regression; *p* < 0. 05 indicates statistical significance. C Heatmap of risk score. D Heatmap of survival status for both groups. E Heatmap showing prognosis-associated lncRNA expression

Univariate Cox analysis was performed on the basis of patients’ clinical features. The findings revealed that age, risk score, stage, and tumour grade were differed greatly and that these characteristics were risk factors for OSCC (Fig. 4A). However, another multifactorial Cox analysis revealed that the risk score may independently predict OSCC prognosis (Fig. 4B) (HR = 1.444, 95% CI = 1.207–1.728).

Ranking of patients according to risk scores to analyse their survival status revealed a lower survival status and higher death likelihood for high-risk patients (Fig. 4C and D). The differential expression profiles for the eight lncRNAs between the two groups are displayed in a heatmap (Fig. 4E), which shows that FIRRE, AC099850.3, and AC090246.1 expression was obviously increased in the high-risk group, whereas that of LINC01305, AL512274.1, MIAT, and AC079921.2 was significantly decreased. Therefore, the risk model’s accuracy in predicting the prognosis of OSCC patients was confirmed.

### Relationship of clinicopathological features with the risk model

**Fig. 5:**
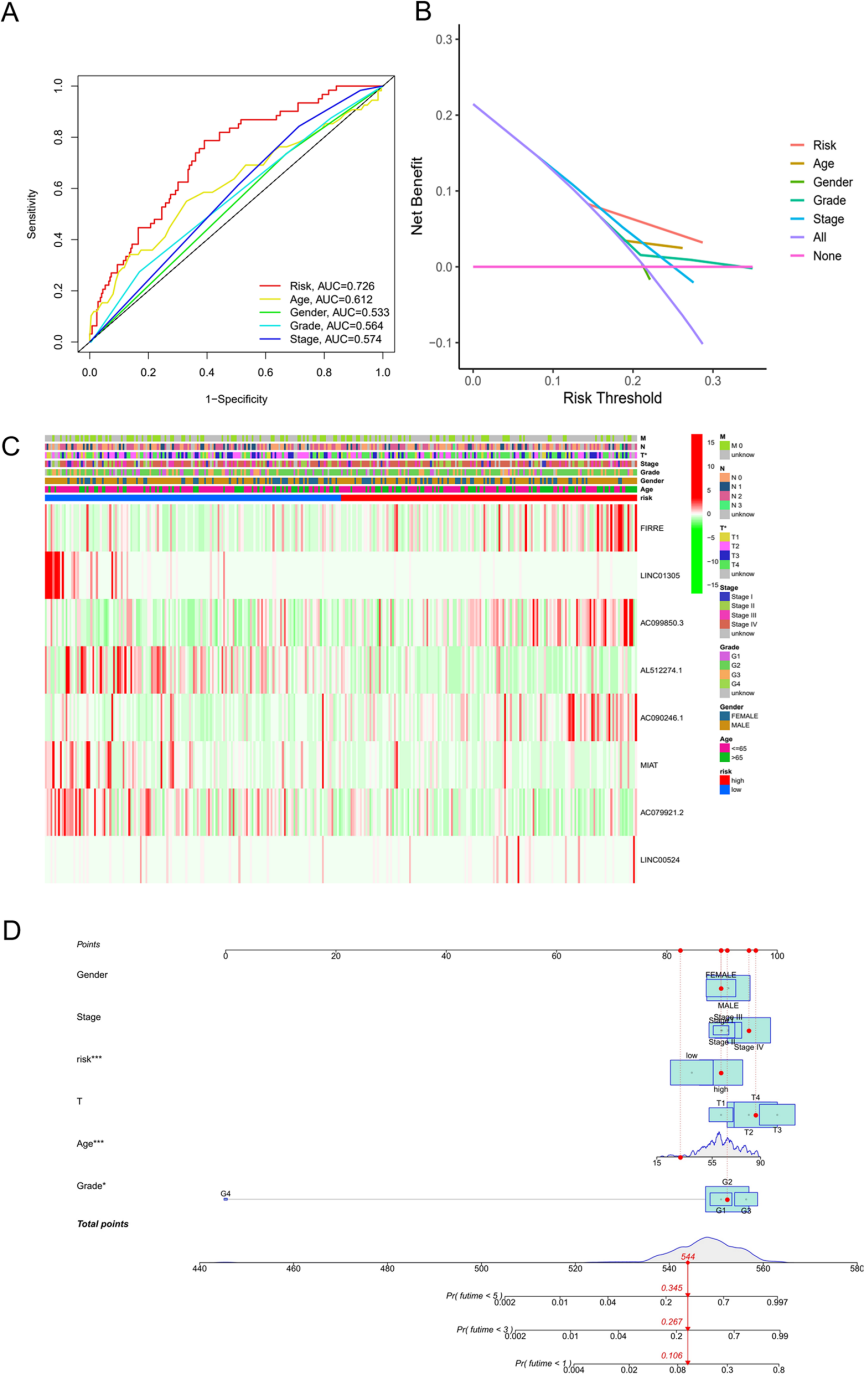
Association of risk model with clinical characteristics. A ROC curves and AUC values for the risk model and clinical indicators. B DCA curves for the risk model and clinical indicators. C Heatmap showing the correlation between prognosis-related lncRNAs and clinical indicators; *p* < 0.05 is considered significantly different. D Prognosis-related column line plot. E Internal calibration curve of the column line graph

To assess the difference in prognosis predicted by the risk model and analysis clinicopathological features, ROC curves of clinical features and risk scores were drawn. As shown in Fig. 5A, the risk model’s AUC value exceeded that of other clinical indicators (AUC = 0.726, 1 year). We then plotted a DCA curve, which indicated that the risk score was a better prognostic factor than other clinical indicators (Fig. 5B). Immediately afterwards, we evaluated the relationship between clinical indicators and risk values for each patient, and the results were plotted in a heatmap (Fig. 5C), which showed a significant difference in the T stage of OSCC of both groups (*p* < 0.05). Subsequently, we constructed a nomogram including age, sex, stage, grade, risk score, TN stage and other prognostic factors with the nomogram’s internal calibration curves. Then, we selected an OSCC patient and used the patient’s data for scoring. Based on the score, the probability of this patient’s surviving less than 1, 3 and 5 years was predicted (the probability of survival less than 1, 3 and 5 years was 8.33, 21.8 and 28%, respectively), and personalized treatment was determined to be an option (Fig. 5D). In addition, the results also showed that the nomogram correction curves at 1, 3 and 5 years were very close to the ideal line, which indicated that the nomogram exhibited high accuracy in predicting the survival rate of the patient at 1, 3 and 5 years (Fig. S1).

**Fig. 6:**
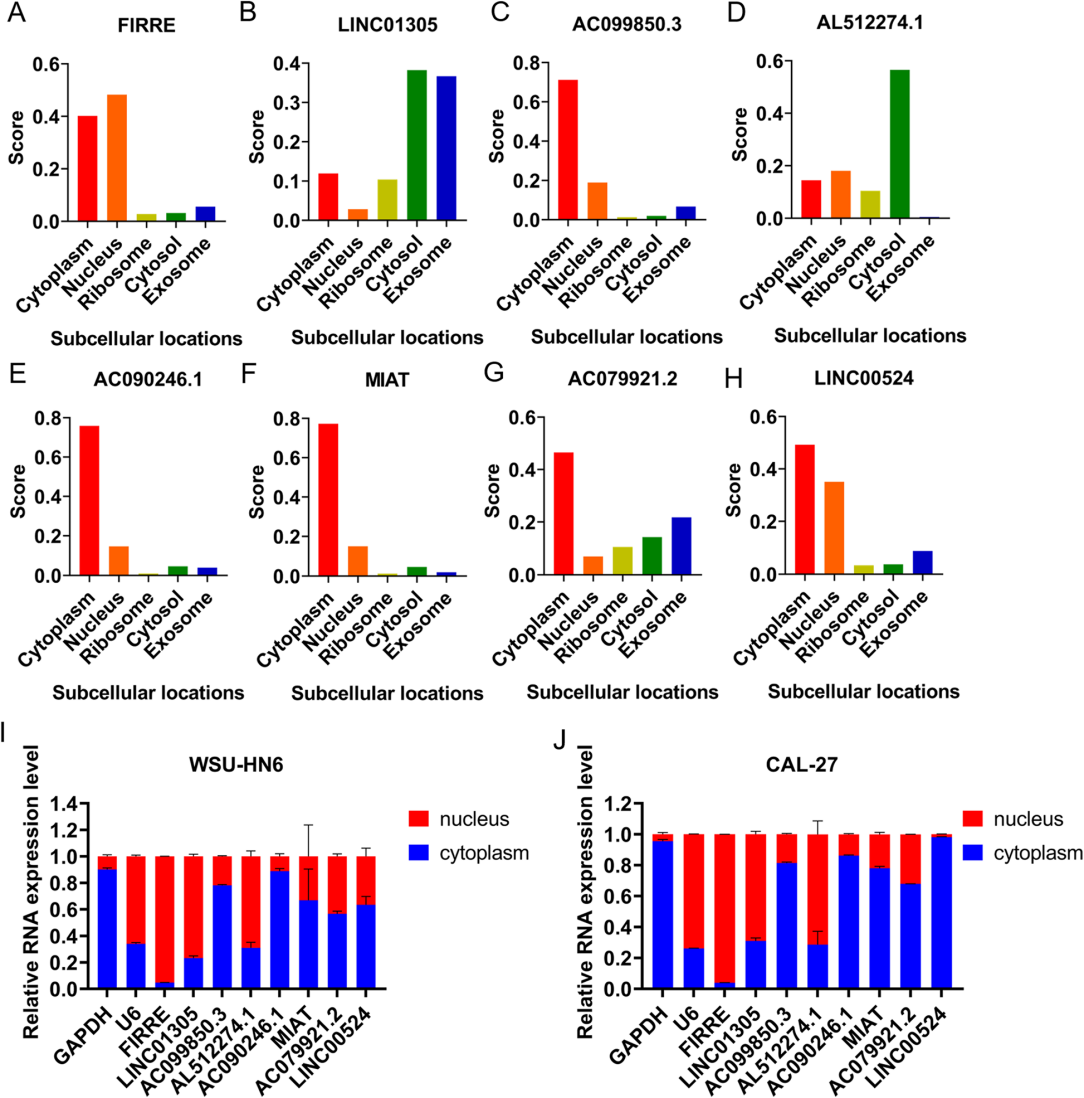
The subcellular localization of eight lncRNAs was predicted using lncLocator (**A**-**H**) and RT-PCR (**I**, **J**)

Moreover, considering that the cellular localization of lncRNAs determines the underlying mechanisms, we analysed the subcellular localization of the eight lncRNAs via lncLocator. As shown in Fig. 6A-H, AC099850.3, AC090246.1, MIAT, AC079921.2 and LINC00524 were mainly located in the cytoplasm, the other two lncRNAs (LINC01305 and AL512274.1) were mainly distributed in the cytosol, and FIRRE was mainly located in the nucleus. Subsequently, the results of in vitro experiments were consistent with the predicted results of the database. In two OSCC cell lines, FIRRE, LINC01305 and AL512274.1 were localized in the nucleus. While, AC099850.3, AC090246.1, MIAT, AC079921.1 and lINC00524 were localized in the cytoplasm (Figs. 6 I, J).

### Differential immune cell infiltration and function between the two groups

**Fig. 7:**
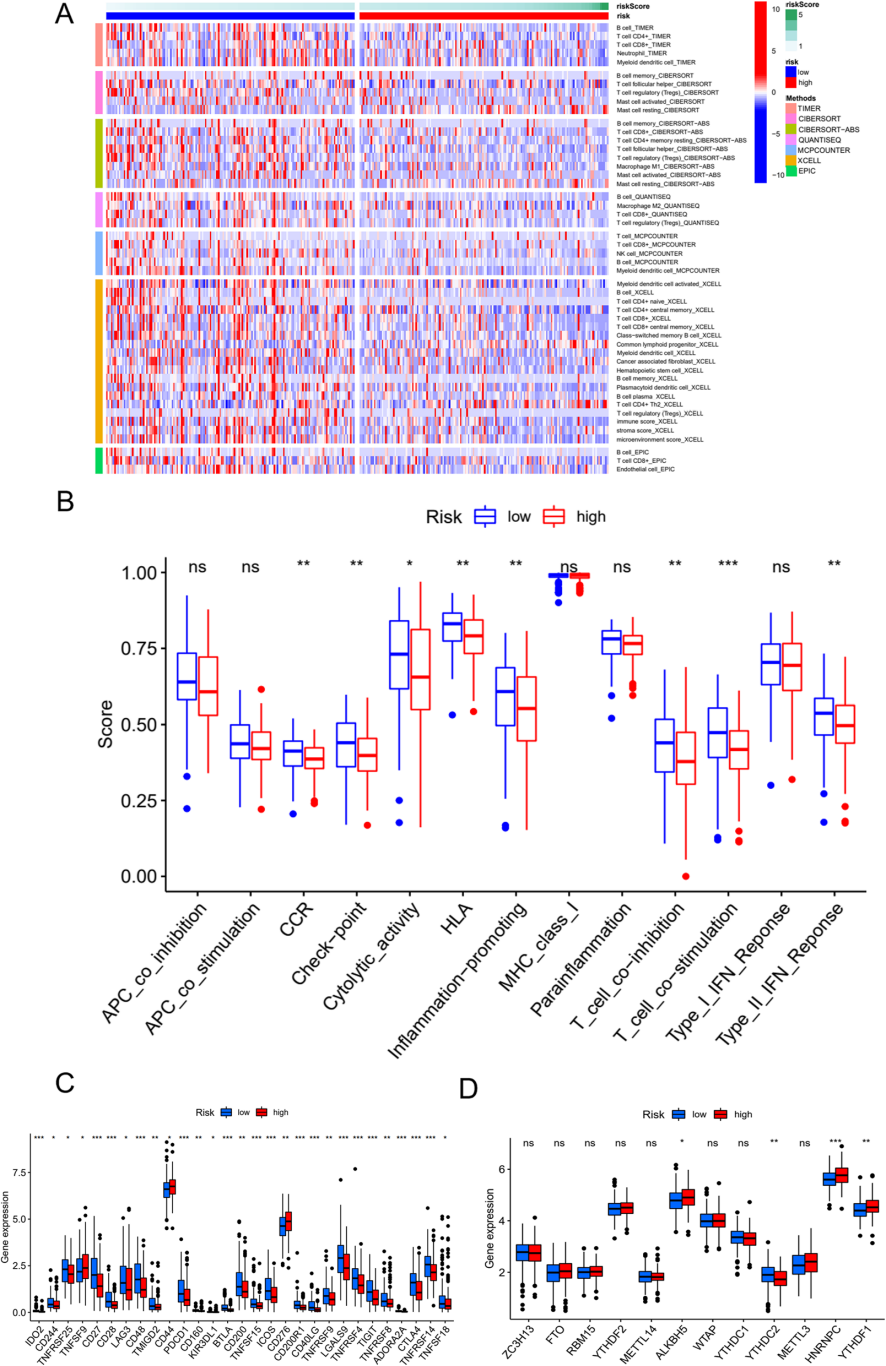
Differences in immune function and immune cell infiltration between the two groups. A Heatmap showing the degree of immune cell infiltration in both groups of OSCC patients. B Comparison of immune function between both groups of OSCC patients. C Differences in ICI expression between both groups of OSCC patients. D Differences in m6A-associated gene expression between the two groups of OSCC patients. *p < 0.05; **p < 0.01; ***p < 0.001

The association of the risk model with immune cell infiltration was explored. The immune cell infiltration analysis results for both groups are presented in a heatmap (Fig. 7A). Vertical coordinates represent immune cell infiltration results for both groups as predicted by different software. Furthermore, immune functions were compared between the two groups, and differences between the groups in immune-related functions, including T-cell costimulation, T-cell coinhibition, CCR, and HLA were identified (Fig. 7B). Thus, the results of the ssGSEA of immune infiltration suggested that immune status was significantly different between the two groups, suggesting a need to develop individualized immunotherapy for OSCC patients.

In addition to differences in immune function and immune cell infiltration, we also examined differences in m6A-associated genes and immune checkpoints between the two groups. A total of 48 immune checkpoints were analysed, and only 29 checkpoint genes were found to be expressed significantly differently between the groups, as shown in Fig. 7C. The expression of m6A-related genes, including ALKBH5, HNRNPC, and YTHDF1, exhibited significant upregulation in high-risk patients (*p* < 0.05), whereas YTHDC2 gene expression was significantly downregulated in high-risk patients (*p* < 0.01) (Fig. 7D).

### Functional analysis

**Fig. 8:**
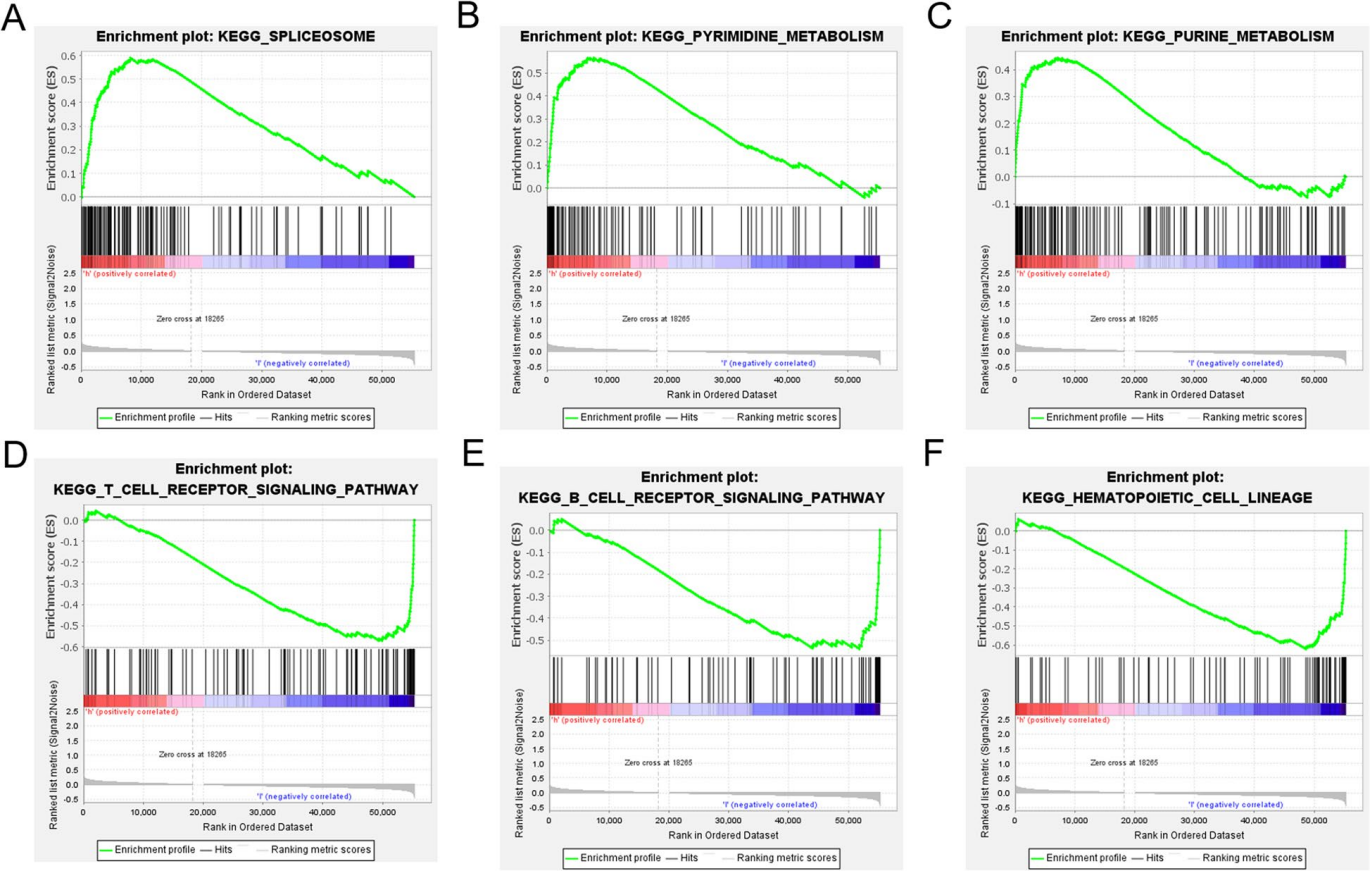
KGEE enrichment plot for high- and low-risk groups. A Spliceosome (*p* < 0.05, ES = 0.589622). B Pyrimidine metabolism (*p* < 0.01, ES = 0.56456). C Purine metabolism (*p* < 0.05, ES = 0.443711). D T-cell receptor signalling pathway (*p* < 0.01, ES = -0.57104). E B-cell receptor signalling pathway (*p* < 0.01, ES = -0.5397). F FcεRI signalling pathway (*p* < 0.001, ES = -0.51729)

The KEGG enrichment analysis was performed to assess differences in the pathways enriched between the two groups. Based on the findings, 10 active pathways were identified in the high-risk patients and as many as 24 active signalling pathways were identified in the low-risk patients (*p* < 0.05). Figure 8 shows the key enrichment results. More active pathways in high-risk patients were related to metabolism, such as spliceosome, pyrimidine metabolism, and purine metabolism. On the other hand, significant enrichment in the low-risk group was identified in immune-associated biological process terms: B-cell receptor pathway, T-cell receptor pathway, and FcεRI pathway.

### L1000FWD analysis led to the identification of potential target drugs

**Fig. 9:**
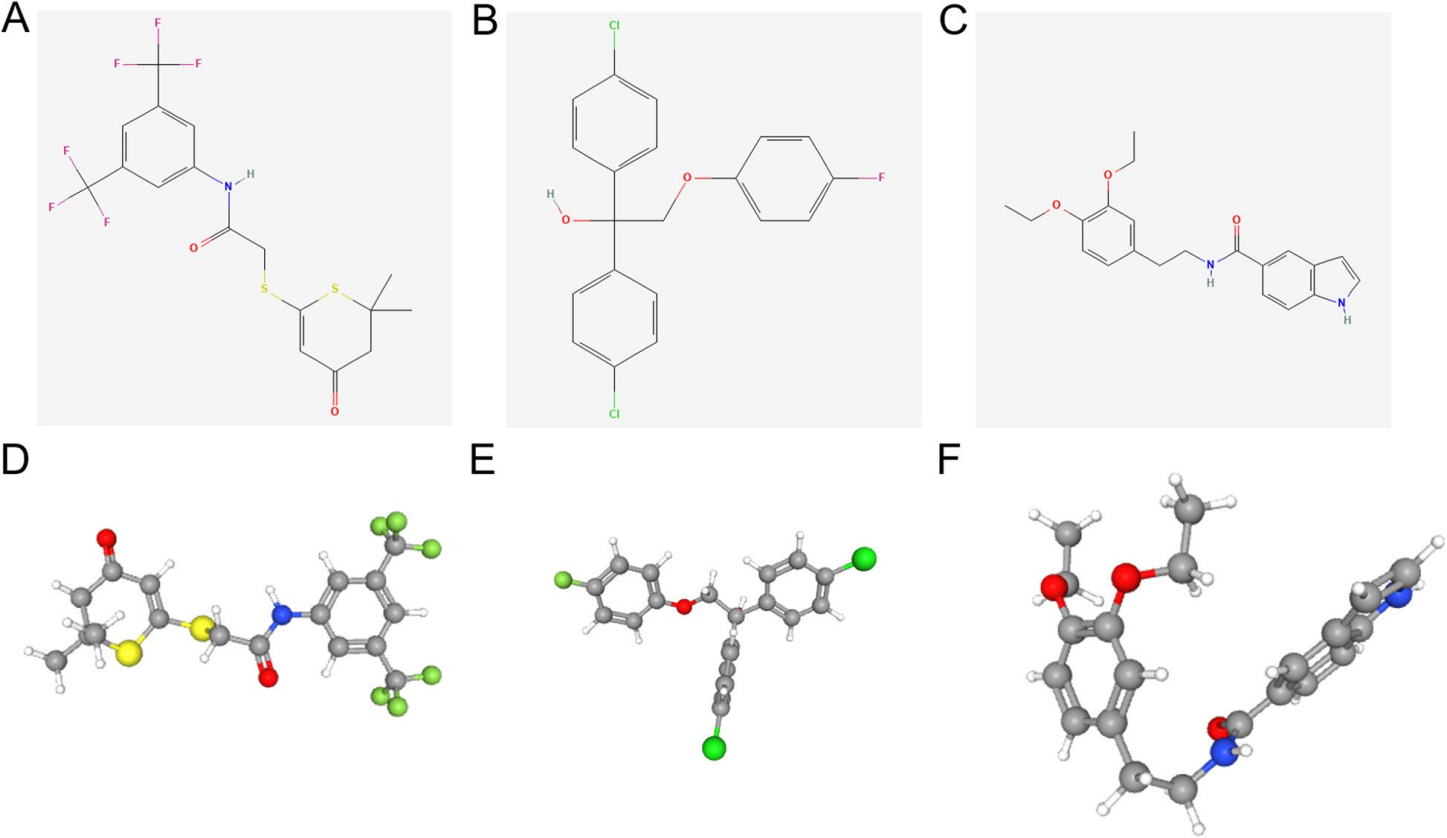
Structures of the top three small-molecule drug candidates. A The 2D structure of KM-03949SC. B The 2D structure of RJC-00245SC. C The 2D structure of BRD-K82185908. D The 3D structure of KM-03949SC. E The 3D structure of RJC-00245SC. F The 3D structure of BRD-K82185908

We searched for potential target drugs in OSCC by uploading the up- and downregulated differentially expressed ferroptosis genes to the L1000FWD database. The top ten drug candidates were obtained, and the basic information of the drugs is shown in Table 2. Treatment with these drugs led to differences in gene enrichment, and thus, MEK inhibitors, oestrogen receptor agonists, RAF inhibitors, etc., were identified Therefore, these small-molecule drugs may be candidate drugs for OSCC treatment and be references for the development of new individualized small-molecule drugs. Among these small-molecule drugs, we selected the three most promising for visualization, and the 2D and 3D structures of KM-03949SC, RJC-00245SC and BRD-K82185908 are shown in Fig. 9.

**Table 2:** Major drugs recognized via use of the L1000FWD database

Rank	Drug	Similarity Score	P-value	Q-value	Z-score	Combined Score	MOA
1	KM-03949SC	-0.4907	1.23e-66	1.06e-62	1.80	-118.88	MEK inhibitor
2	RJC-00245SC	-0.4352	1.37e-53	1.15e-50	1.73	-91.41	estrogen receptor agonist
3	BRD-K82185908	-0.4352	1.99e-51	1.29e-48	1.75	-88.74	adrenergic receptor antagonist
4	KM-00519SC	-0.4259	3.78e-52	2.53e-49	1.72	-88.55	RAF inhibitor
5	BRD-K94987138	-0.4167	6.71e-51	3.99e-48	1.73	-86.64	histamine receptor antagonist
6	BRD-K67619794	-0.4074	3.33e-49	1.53e-46	1.85	-89.77	histamine receptor antagonist
7	BRD-K05197617	-0.4074	5.57e-49	2.46e-46	1.73	-83.71	EGFR inhibitor
8	Ivermectin	-0.3611	7.32e-40	1.20e-37	1.87	-73.29	benzodiazepine receptor agonist
9	Vemurafenib	-0.3426	1.96e-36	2.20e-34	1.81	-64.68	RAF inhibitor
10	BRD-K03122949	-0.3426	1.44e-36	1.64e-34	1.74	-62.28	dopamine receptor antagonist

### Internal validation and real-time PCR

**Fig. 10:**
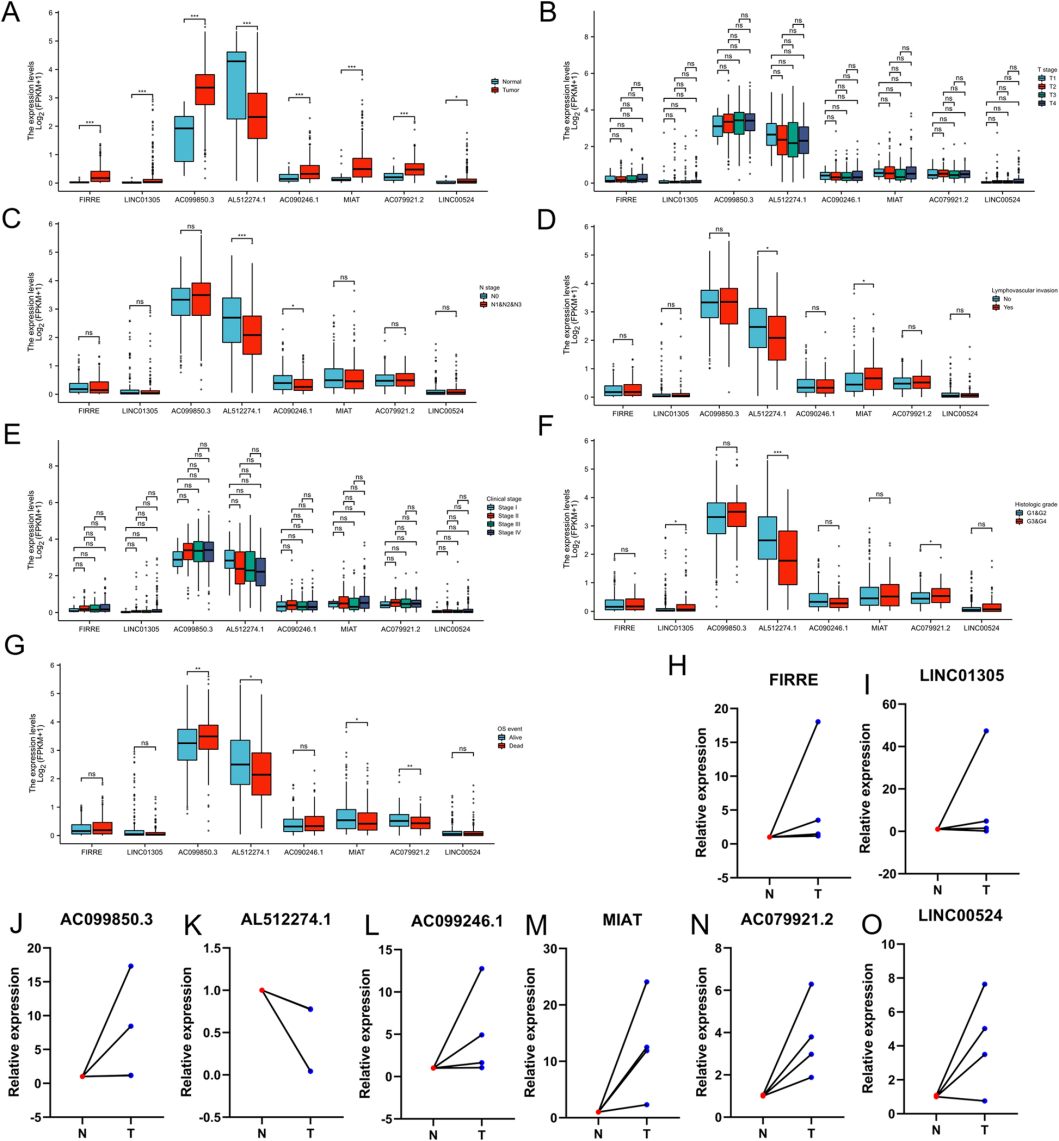
Internal validation and real-time PCR. A Expression of 8 lncRNAs in TCGA. B-G Correlation of lncRNAs with T stage, N stage, lymphovascular invasion, Stage, Grade and OS event, respectively. H–O Expression of lncRNAs in four pairs of matched OSCC (T), adjacent normal tissues (N)

We also analysed differences in the expression of ferroptosis-related lncRNAs with respect to different clinical features (Figs. 10A-G). FIRRE, LINC01305, AC099850.3, AL512274.1, AC090246.1, MIAT, AC079921.2 and LINC00524 were differentially expressed in tumour and normal tissues (Fig. 10A). In addition, AL512274.1 and AC090246.1 was differentially expressed in N stage tumours (Fig. 10C); AL512274.1 and MIAT were differentially expressed in lymphovascular invasion (Fig. 10D); LINC01305, AL512274.1 and AC079921.2 were differentially expressed in different grades (Fig. 10F); and AC099850.3, AL512274.1 and MIAT expression was strongly correlated with OS events in OSCC patients (Fig. 10G). In addition, we also detected the expression levels of eight lncRNAs in four pairs of matched OSCC (T), adjacent normal tissues (N). As shown in Figs. 10H-O, the relative expression levels of FIRRE, LINC01305, AC099850.3, AC090246.1, MIAT, AC079921.2 and LINC00524 in OSCC tissues were higher than those in adjacent normal tissues, while the relative expression level of AL512274.1 was lower than those in adjacent normal tissues. Therefore, the expression levels of the eight lncRNAs were consistent with the results of our model analysis.

## Discussion

Patients with OSCC, a common head and neck cancer, have an overall poor prognosis. According to the latest NCCN dental guidelines, surgery, chemotherapy and radiotherapy are recommended for OSCC. Through individualized therapy, treatments are selected on the basis of different states of the disease [30]. In recent years, the use of multidisciplinary therapies has enabled OSCC patients to obtain optimal treatment options with minimal risk of complications. A pathologist is responsible for a definitive diagnosis, a surgeon completely removes the lesion, and a radiologist performs precision radiotherapy. In addition to advising on postoperative repair and care, our goal is to defeat OSCC [31]. In this process, an effective predictor of OSCC prognosis is critical for treatment decisions. However, the most commonly used TNM staging and relevant clinical features of patients lead to certain limitation in the analysis [32,33]. TNM staging is based solely on the number and size of positive lymph nodes and does not pay account for negative lymph node number. Therefore, an increasing number of studies has emphasized the importance of negative lymph node and total lymph node numbers in the prognosis of OSCC. These two indicators have been shown to be independent prognostic factors in several malignancies [34–36]. A retrospective case analysis of 120 patients with OSCC in Europe indicated that there were insignificant differences in prognosis between young patients and patients 40 years or older [37]. A similar retrospective analysis from Taiwan did not reveal a difference in survival between males and females with OSCC [38]. These results suggested that TNM stage and clinical features are not ideal factors for predicting OSCC prognosis. Therefore, it is necessary to find other prognostic indicators to accurately determine the prognosis and guide treatment for patients. In this research, a bioinformatics method was adopted to explore the effect of ferroptosis-related lncRNAs on OSCC prognosis. A prognostic risk model was established for validating model accuracy, which offered a new perspective on the effect of ferroptosis on OSCC pathogenesis and prognosis.

The prognostic model developed in this study included eight ferroptosis-related lncRNAs (FIRRE, LINC01305, AC099850.3, AL512274.1, AC090246.1, MIAT, AC079921.2, and LINC00524). FIRRE is related to breast cancer [39], gallbladder cancer [40] and diffuse large B-cell lymphoma [41]. In another study, FIRRE inhibited proinflammatory factor production by decreasing the expression of HMGB1, thus relieving neuropathic pain in female rats [42]. In oesophageal squamous carcinoma, LINC01305 was found to regulate HTR3A mRNA, thereby promoting cancer cell metastasis and proliferation [43]. In addition, LINC01305 regulated the epithelial-mesenchymal transition (EMT) in cervical cancer [44] and lung cancer [45] through different pathways. MIAT is also an important lncRNA that was first found to be associated with myocardial infarction [46] and later it was found to be involved in the progression of tumours, including retinoblastoma [47], smooth muscle tumour [48], and nasopharyngeal carcinoma [49]. AC099850.3, AL512274.1, AC090246.1, AC079921.2, and LINC00524 have rarely been studied in solid tumours, but different bioinformatics analysis methods suggest their potential prognostic value in different cancers (AC099850.3 [50] in non-small-cell lung cancer, AL512274.1 [51] in OSCC, and LINC00524 [52] in clear cell renal cell carcinoma). Thus, the eight lncRNAs identified in our study are coexpressed with many ferroptosis genes, and they are previously identified lncRNAs. Unfortunately, they have not been extensively studied in OSCC, and their biological functions in OSCC have not been reported.

Ferroptosis is inextricably linked to cellular metabolism. When the dynamic balance of cellular metabolism in the body is disrupted, ferroptosis leads to many diseases caused by metabolic imbalance, such as heart disease and brain injury [53]. By performing a functional enrichment analysis, we found that the pathways associated with high-risk cases were primarily cellular metabolism-related pathways such as purine metabolism, pyrimidine metabolism, and amino acid metabolism, indicating that ferroptosis is closely related to cellular metabolism in OSCC, which aligns with the results reported in the literature [53].

Immune cell infiltration results demonstrated that the prognostic model correlated with immune activity. Numerous investigations have been conducted to explore the correlation of ferroptosis with tumour immunity, but these findings were insufficient to determine the exact relationship between the immune system and ferroptosis. The pathways associated with low-risk cases were primarily T and B-cell receptor (TCR, BCR) pathways. TCR is the molecule on the T-cell surface that specifically recognizes antigens and determines the diversity of T cells [54,55]. Signal transduction initiated by T-cell antigen receptors is at the core of T-cell activation, which is necessary for acquired immunity [56]. CD4^+^ T cells and CD8^+^ T cell activation initiates the TCR signalling pathway, allowing the body to respond with the appropriate immune response [57]. BCR is a molecule on the surface of B cells that specifically recognizes antigens, and the activation signal generated by its binding to antigens is the first signal in B-cell activation by regulating B-cell gene transcription. These results suggest that the acquired immune system is more active and immune activity is stronger in low-risk OSCC patients than in high-risk OSCC patients. Additionally, differences in immune checkpoint, immune function, and m6A-associated genes between the two groups offer a theoretical foundation for the development of individualized immune-targeted therapies for OSCC patients. Small-molecule drugs predicted on the basis of ferroptosis-related DEGs have not yet been experimentally validated, and their specific role in OSCC still needs further exploration.

Although we constructed a highly accurate prognostic model for OSCC patients, it must be admitted that there were still some limitations in this study. This model was derived from the comprehensive genome analysis of OSCC cases in TCGA database, and lacked the ability to specifically recognize tumor cells. However, OSCC was known to be highly heterogeneous and to have a poor prognosis [58]. This heterogeneity can occur not only among individuals, but also within the same individual or even within the same organization. Therefore, further study on heterogeneity is necessary. Our model needs to be combined with single-cell sequencing to better understand the heterogeneity between cells, and its applicability and accuracy also need to be further explored in clinical patients with OSCC. In conclusion, the prognostic model of ferroptosis-related lncRNAs was expected to be a novel biomarker for OSCC diagnosis and treatment decision making. However, further in vitro and in vivo studies are needed to clarify the role and mechanism of ferroptosis-related lncRNAs in the OSCC occurrence and development.

## Conclusions

We constructed a new prognostic model of OSCC based on ferroptosis-related lncRNAs. The model is valuable for prognostic prediction and immune evaluation, laying a foundation for the study of ferroptosis-related lncRNAs in OSCC.

## Supplementary Information

**Additional file 1**: **SupplementaryFigure 1.** Internal calibrationcurve of column line graph. **Supplementary Table 1:** The primer sequences of lncRNAs. 

## Data Availability

This study followed the policies and guidelines for data access and publication specified by The Cancer Genome Atlas (TCGA) database (https://portal.gdc.cancer.gov/). The public databases involved in this research are as follows: FerrDb website (http://www.zhounan.org/ferrdb/), LNCipedia (https://lncipedia.org/), lncLocator database (http://www.csbio.sjtu.edu.cn/bioinf/lncLocator/).

## Abbreviations

lncRNAs: Long non-coding RNAs
OSCC: Oral squamous cell carcinoma
TCGA: The Cancer Genome Atlas
GDC: Genome Data Sharing
LASSO: Least absolute shrinkage and selection operator
ROC: Receiver Operating Characteristic
DCA: Decision Curve Analysis
AUC: Areas under the curve
TME: Tumor microenvironment
OS: Overall survival
KEGG: Kyoto Encyclopedia of Genes and Genomes
TCR: T cell receptor
BCR: B cell receptor; EMT: epithelial-mesenchymal transition
