# Supplementary material for: Potential prognostic value of a eight ferroptosis-related lncRNAs model and the correlative immune activity in oral squamous cell carcinoma
Source: BMC Genom Data. 2022 Nov 16;23:80. doi: 10.1186/s12863-022-01097-z (PMC9667687; doi:10.1186/s12863-022-01097-z)
Supplement: Supplementary file 1 — Additional file 1: SupplementaryFigure 1. Internal calibrationcurve of column line graph. Supplementary Table 1: The primer sequences of lncRNAs. [file 12863_2022_1097_MOESM1_ESM.docx]

**Supplementary Materials**


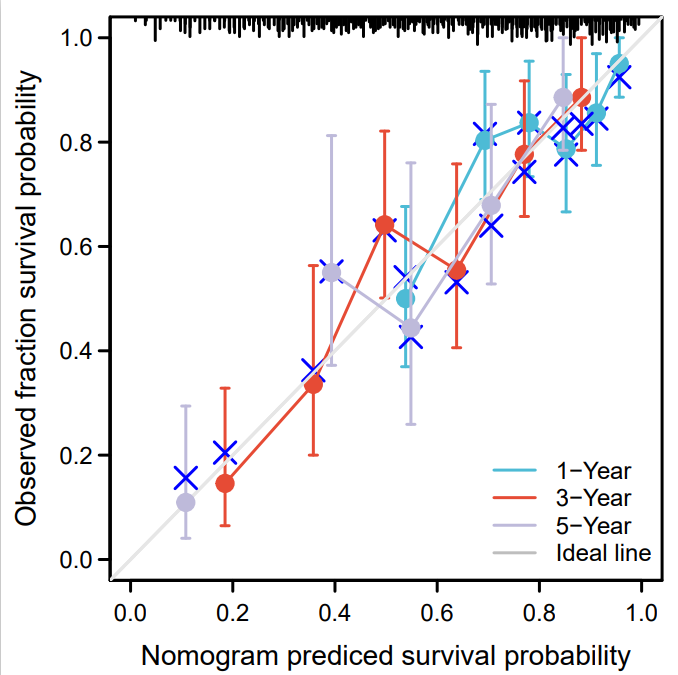


Supplementary Figure 1 Internal calibration curve of column line graph.

Supplementary Table 1: The primer sequences of lncRNAs.

| FIRRE-F | TCAGGGAGAATTGGTCTGCC |
| --- | --- |
| FIRRE-R | GAGGTCCACAGCAAGCATACAT |
| LINC01305-F | ACGTAATTAGGAGGCCACGC |
| LINC01305-R | GTCGCCCAAACTCAGGCTTA |
| LINC00524-F | GACCATCAACCTCGGAAGGC |
| LINC00524-R | GAAAGAGGAGATGCGTGGGC |
| AC090246.1-F | TCTTTGCTACTGGTTGCTGGG |
| AC090246.1-R | AGCACAACCACTTACGAACCAT |
| AC079921.2-F | ATGAGGCCTCCGAGAGGATG |
| AC079921.2-R | TCCACCTGGGATCCTCTGTC |
| MIAT-F | CCCACATTCTTCCTCCGTCT |
| MIAT-R | ACACACACAGGAGTCGCTAA |
| AC099850.3-F | TCGCTATGTTTCCCAGGCTGTATT |
| AC099850.3-R | TGCCAAGGAATCTCTGAAGTCCAT |
| AL512274.1-F | AGAGGCAGCTCACATTCACT |
| AL512274.1-R | GGATCGTGCCTGGTACCTAA |
